# Unsuccessful Attempt at Poisoning with Pounded Glass

**Published:** 1849-01

**Authors:** 


*Unsuccessful attempt at poisoning with Pounded Glass.*—We make the following extract of a letter from our intelligent correspondent, W. K. Bowling, M. D., of Adairville in this State, dated Oct. 15th, 1848.

"Mrs. C. of this village, in her attentions to her child 9 months of age, after a discharge from its bowels, discovered some particles o- glass adhering to its nates. Becoming alarmed she sent for my partf ner, Dr. Poor, who, upon his arrival, had the fæces washed, and procured more than a tea-spoonful of powdered glass. He gave the child a dose of castor oil, and superintended in person the washing of the discharges as long as any glass was found in them and procured by weight *eighty grains!* The glass had been irregularly powdered and exhibited fragments of every size from a grain of wheat to the finest sand. The child showed not the slightest indisposition, and remains perfectly well up to the present time, (five days) since the last glass was discovered in its discharges.

I have thought this case worthy of preservation for two reasons : 1st. Because physicians rarely have an opportunity of witnessing the effecf of pulverized glass upon the gastro-intestinal mucous membrane ot man. 2d. Because the case appears to demonstrate that this substance does not exercise any deleterious influence."—*West. Journ. of Med. and Surg.*

